# PPARα siRNA–Treated Expression Profiles Uncover the Causal Sufficiency Network for Compound-Induced Liver Hypertrophy

**DOI:** 10.1371/journal.pcbi.0030030

**Published:** 2007-03-02

**Authors:** Xudong Dai, Angus T. De Souza, Hongyue Dai, David L Lewis, Chang-kyu Lee, Andy G Spencer, Hans Herweijer, Jim E Hagstrom, Peter S Linsley, Douglas E Bassett, Roger G Ulrich, Yudong D He

**Affiliations:** 1 Informatics, Rosetta Inpharmatics, Seattle, Washington, United States of America; 2 Preclinical Molecular Profiling, Rosetta Inpharmatics, Seattle, Washington, United States of America; 3 Mirus Bio Corporation, Madison, Wisconsin, United States of America; 4 Cancer Biology, Rosetta Inpharmatics, Seattle, Washington, United States of America; Amgen, United States of America

## Abstract

Uncovering pathways underlying drug-induced toxicity is a fundamental objective in the field of toxicogenomics. Developing mechanism-based toxicity biomarkers requires the identification of such novel pathways and the order of their sufficiency in causing a phenotypic response. Genome-wide RNA interference (RNAi) phenotypic screening has emerged as an effective tool in unveiling the genes essential for specific cellular functions and biological activities. However, eliciting the relative contribution of and sufficiency relationships among the genes identified remains challenging. In the rodent, the most widely used animal model in preclinical studies, it is unrealistic to exhaustively examine all potential interactions by RNAi screening. Application of existing computational approaches to infer regulatory networks with biological outcomes in the rodent is limited by the requirements for a large number of targeted permutations. Therefore, we developed a two-step relay method that requires only one targeted perturbation for genome-wide de novo pathway discovery. Using expression profiles in response to small interfering RNAs (siRNAs) against the gene for peroxisome proliferator-activated receptor α (*Ppara*), our method unveiled the potential causal sufficiency order network for liver hypertrophy in the rodent. The validity of the inferred 16 causal transcripts or 15 known genes for PPARα-induced liver hypertrophy is supported by their ability to predict non-PPARα–induced liver hypertrophy with 84% sensitivity and 76% specificity. Simulation shows that the probability of achieving such predictive accuracy without the inferred causal relationship is exceedingly small (*p* < 0.005). Five of the most sufficient causal genes have been previously disrupted in mouse models; the resulting phenotypic changes in the liver support the inferred causal roles in liver hypertrophy. Our results demonstrate the feasibility of defining pathways mediating drug-induced toxicity from siRNA-treated expression profiles. When combined with phenotypic evaluation, our approach should help to unleash the full potential of siRNAs in systematically unveiling the molecular mechanism of biological events.

## Author Summary

Approaches for discovering mechanisms of action and for identifying molecular biomarkers in biomedical research are evolving today, as the growing symbiosis with computational sciences becomes more widely appreciated. In fact, the combination of various new technologies has been pushing forward both frontiers. Here, we present an example of the combined use of in vivo siRNA knock-down technology, genome-wide gene expression profiling, and computational reasoning to unveil regulatory causal relationships and the sufficiency network of identified genes for compound-induced toxicity. Unlike previously reported approaches, our method requires only one targeted perturbation for genome-wide de novo pathway discovery. Hence, our method can be directly applied to animal models in which it is still technically challenging to perform genome-wide genetic perturbation or RNAi screening. The independent application of our derived model to compounds with unrelated mechanisms of action suggests the existence of a universal molecular module that mediates liver hypertrophy. The resulting sufficiency network for induction of liver hypertrophy will have an immediate impact on the progress of toxicogenomics. When combined with phenotypic evaluation, our approach should help to unleash the full potential of siRNAs in systematically unveiling the molecular mechanisms of biological events.

## Introduction

Determining the pathways underlying drug-induced toxicity from genome-wide expression profiles is essential for drug development. Knowledge of the components of the identified pathways and their sufficiency order for toxicity is crucial for selecting mechanism-based biomarkers to assess the safety of novel agents. Numerous efforts have been made to establish toxicogenomics databases, most of which consist of thousands of expression profiles produced in response to treatment with dozens or hundreds of compounds and their associated toxicities [1–5]. Classifiers based on genes showing altered expression in association with the observed toxicities have been developed to predict a variety of potential toxicities in target tissues, such as liver, kidney, and heart [1]. Although these metrics built on different machine learning algorithms could be useful in estimating the severity of potential toxicities induced by compounds, the applications of these classifiers in understanding the mechanisms of drug-induced toxicity are not straightforward. In particular, this approach is unlikely to distinguish the upstream causal genes from the downstream responsive genes among all the genes associated with an induced toxicity. Without knowledge of the causal sufficiency order, designing experiments to test predicted toxicity in animal models remains difficult.

Genome-wide RNA interference (RNAi) has emerged as an effective tool in uncovering components of pathways for specific cellular functions and biological activities, such as the innate immune response, the DNA-damage response, Golgi organization, and lifespan regulation in *Saccharomyces cerevisiae, Caenorhabditis elegans,* and *Drosophila* [6–12]. However, eliciting the causal sufficiency relationships among identified genes for a biological activity is still challenging. Phenotypic screening by RNAi has successfully identified regulators of a gene on a single-gene basis [13]; however, it is unrealistic to exhaustively examine all potential interactions by concomitant perturbations targeting each gene within a pathway, given the exponential number of potential interactions between these genes and the phenotype [6–12]. Therefore, a high-throughput approach to identify the most likely sufficiency order among these genes is critical for the application of RNAi screening in defining phenotype-orientated pathways. Such an approach is particularly important for pathway discovery in rodents, the most frequently used animal models in preclinical studies. Unfortunately, introducing genome-wide targeted genetic modifications in rodents is technically challenging.

Computational approaches have been reported to infer regulatory networks from expression profiles [14–20]. A variety of statistical modeling approaches, including Bayesian-based networks, graph theory, and ordinary differential equations, have been used for depicting or uncovering molecular networks. Collins and colleagues first developed a model for identifying the mode of action for a compound after perturbing each of the genes in the network [17]. Friedman and colleagues reported a Bayesian network approach for de novo inference of potential transcriptional networks based on 300 expression profiles obtained from yeast strains with 276 deletion mutations [18]; this approach did not require prior knowledge about the modeled network, as Collins' approach did. Approaches based on physical interactions, such as DNA–protein or protein–protein interactions, were reported by Yeang et al., Gifford, and a couple of other groups as well [20,21]. Although implemented successfully in *S. cerevisiae,* these published approaches depend on the accessibility of genome-wide targeted perturbations in individual model systems. Even in models in which genome-wide perturbation by genetic engineering can be easily achieved, such as yeast or fly models, it is still quite labor intensive and prohibitively expensive to use a genome-wide overexpression or deletion mutation to obtain the data required for these analytic approaches. In rodents, it is unlikely that such a dataset from genome-wide genetic permutation could be obtained with current technology. Some progress has been made with the chromatin immunoprecipitation (ChIP)–chip technology for eliciting potential transcriptional regulatory relationships; however, screening for all the transcription factors on the genome scale is impeded by the lack of specific antibodies for each of the known transcription factors.

Recent work by Segal and colleagues in identifying regulatory modules from five strains of yeast shows promise [14,15]. Using precompiled putative transcription factors, they identified potential transcriptional modules that are associated with enriched functionality, suggesting the potential impact of these genes on certain biological processes. Others have attempted to associate the derived network with specific biological processes as well [18,22–24]. Recent progress toward identifying a phenotype-oriented network in rodent models was initially reported by Schadt et al. [23]. Taking advantage of the genetic heterogeneity introduced by more than 300 crossbreeding F2 mice, the authors inferred causal genes for obesity from a combination of genetic quantitative trait loci and their associated expression profiles. The sensitivity and specificity of their approach depend on three factors: the frequency of recombination across the genome, the density of the measured genetic markers, and the coverage of the phenotypic alteration of interest among the F2 subpopulations. To ensure adequate statistical power, their approach requires large-scale cross-breeding and genotyping, yet it may not be flexible enough for inferring causal genes for drug-induced toxicities in any target tissue.

To uncover a phenotype-orientated network for any type of drug-induced toxicity in rodents, we developed a two-step relay approach to infer causal genes and their sufficiency order from small interfering RNA (siRNA) response expression profiles. In the first step, we used both the on- and off-target effects from RNAi to infer pairwise regulatory relationships from the expression profiles. This strategy allowed us to avoid the need for large-scale targeted genetic perturbations required by the majority of previously developed methods. In the second step, we used the inferred regulatory relationships among the identified gene pairs to derive causal genes for any type of drug-induced toxicity from any previously established toxicogenomics database in the rodent. Our approach provides a practical and flexible strategy for uncovering potential molecular mechanisms for drug-induced toxicity.

## Results

### Inferring Pairwise Regulatory Relationships from siRNA-Treated Expression Profiles

**Figure 1:**
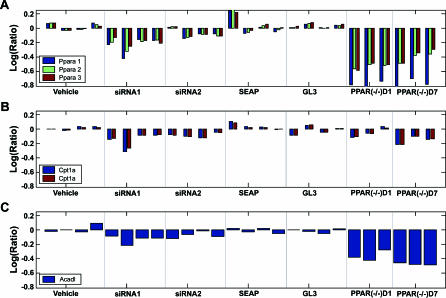
Effective Modification of *Ppara* mRNA and Its Known Target Genes by siRNA In Vivo *Ppara* was monitored with three probes, *Ppara* 1, 2, and 3, targeting different fragments of the whole *Ppara* transcript. The mRNA levels of *Ppara,* as detected by all three *Ppara* probes (A), and by its known target genes *Cpt1a* (B) and *Acadl* (C) decreased coordinately in the livers of mice treated with *Ppara* siRNA1 and *Ppara* siRNA2. The mRNA levels of *Ppara* and its known target genes, *Cpt1a* or *Acadl,* were also measured in *Ppara* ^−/−^ mice as a positive control for the silencing effect of *Ppara* siRNA. Similar concordant regulation between *Ppara* and its known targets is observed in *Ppara* ^−/−^ mice when compared with wild-type mice at day 1 (D1) or day 7 (D7) after vehicle treatment. The mRNA level for each mouse is indicated by a vertical bar.

To derive causal genes and their sufficiency order in inducing PPARα activation–induced liver hypertrophy (AILH), we profiled mouse liver after modifying *Ppara* mRNA levels by treatment with two *Ppara* siRNAs [25]. The mRNA levels of *Ppara* and two of its known downstream targets, carnitine palmitoyltransferase 1A *(Cpt1a)* and long-chain acyl-CoA dehydrogenase *(Acadl),* were examined in the siRNA-treated mice as well as in *Ppara* knockout mice, which served as a positive control for the silencing efficacy of the *Ppara* siRNAs (Figure 1A). Expression of both *Ppara* and its known target genes decreased in a coordinated fashion among all *Ppara* siRNA–treated mice, and regulation between *Ppara* and its targets, *Cpt1a* and *Acadl,* was confirmed in the *Ppara* knockout mice. These data suggest that *Ppara* siRNA effectively downregulates transcription of the targeted gene, *Ppara,* and its known downstream genes in vivo. The genome-wide expression profiles in livers from the *Ppara* siRNA–treated mice also revealed an extensive off-target effect when compared with the profiles in livers from the *Ppara* knockout mice, as reported separately [26]. A large number of genes that were downregulated in the *Ppara* siRNA–treated liver profiles either were not downregulated or were upregulated in the *Ppara* knockout liver profiles, and vice versa. Hence, *Ppara* siRNA, delivered by tail vein, has a significant effect on both on- and off-target genes in vivo. The transcriptional response to *Ppara* siRNA treatment provides an ideal dataset for inferring pairwise regulatory relationship genes by local constraint algorithm (LCA) in that the external perturbation, or the instrumental variable, on the system can be measured.

**Figure 2:**
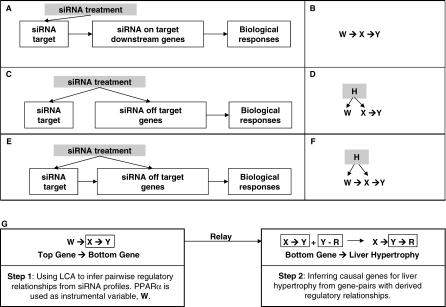
Schematic of the LCA That Determines Genes Mediating Certain Cellular Responses from Expression Profiles after Perturbation by siRNA The three major scenarios for the effect of siRNA on gene expression and biological response are illustrated in (A): the direct target or the on-target gene for siRNA and/or its downstream regulated genes resulting in a cellular response; (C): the off-target genes and their downstream regulated genes causing the cellular response; and (E): the cellular effect of an siRNA toward on-target and/or off-target genes and their downstream regulated genes leading to the cellular response. The corresponding causal models among the instrumental variable *W,* the top gene *X,* and the bottom gene or response *Y* for these scenarios are shown in (B,D,F). The hidden variable, *H,* is not measurable by expression profiles. The flow chart for two-step relay is illustrated in (G).

Figure 2 summarizes the three major causality relationships for the impact of siRNA on PPARα, PPAR downstream genes, PPAR siRNA off-target genes, and cellular responses. The causal models corresponding to all illustrated scenarios and their combinations share a common regulatory relationship between *X* and *Y* (designated as *X* → *Y*), representing genes and/or cellular responses affected by the perturbation variable, *W,* or a hidden variable, *H,* which is not measurable in the current system (Figure 2B, 2D, and 2F). Because the mRNA levels for both the on- and off-target genes are controlled by the siRNA, we considered the effect of *Ppara* siRNA as the instrumental variable (*W*), which is manifested and can be measured by the mRNA level of *Ppara* in response to the siRNA treatments. Without genome-wide perturbation induced by the off-target effect of *Ppara* siRNA, expression of the genes associated with *Ppara,* such as gene *X* and gene *Y,* in the *Ppara* siRNA–treated profiles should highly correlate with expression of *Ppara*. Hence, it would be impossible to determine the regulatory relationship between gene *X* and gene *Y*. However, the off-target effects of different *Ppara* siRNAs often introduce additional perturbations on *X* and *Y* differentially, making it possible to derive the regulatory relationship between gene *X* and gene *Y*. For example, when two conditions—(1) the correlation between *Ppara* and gene *Y* is disrupted by the off-target effects, but the correlation between *Ppara* and gene *X* remains in some profiles, and (2) the disruption of the correlation between *Ppara* and gene *X* is parallel to the disruption of correlation between *Ppara* and gene *Y* in the other profiles—are met simultaneously for *Ppara*, gene *X,* and gene *Y,* then the observation, which statistically satisfies the *Ppara* and *Y* conditional independency given *X,* indicates that it is more likely that *X* regulates *Y* than that *Y* regulates *X*. Consequently, when *W,* an instrumental variable whose value is controlled by a defined input or signal, is known, the causal relationship between *X* and *Y* can be inferred computationally [27,28]. More comprehensive statistical tests, such as the LCA, to estimate the likelihood of *X* regulating *Y* given an instrumental variable *W* over all other possible relationships among *X, Y,* and any other hidden variables, have been previously established for general data-mining purposes [27]. Our two-step relay approach used LCA to infer all pairwise causal relationships between a top gene (*X*) and a bottom gene (*Y*) in the first step, and a causal gene (*X*) and its cellular response (*Y*) in the second step.

**Figure 3:**
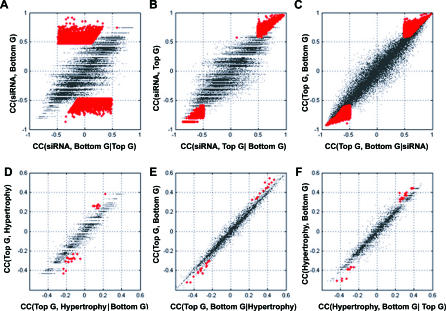
Identification of the Top and Bottom Gene Pairs and the Causal Genes for PPARα–AILH by the Two-Step Relay Approach Conditional independency between the instrumental variable *(Ppara)* and the bottom gene given the top gene in selected gene pairs (A–C) is illustrated. When conditioned on the identified top genes, indicated in red (A), the correlation between siRNA effect, manifested by *Ppara* mRNA level and the bottom genes, vanishes. However, when conditioned on the bottom genes, indicated in red (B), the correlation between *Ppara* and the selected top genes remains significant. Similarly, the dependency between the top genes and liver hypertrophy is abolished when conditioned on the bottom genes (*p* < 0.01), indicated by red dots in (D). The dependency between the top genes and bottom genes remains when conditioned on liver hypertrophy (E), as does the dependency between liver hypertrophy and the bottom genes (*p* < 0.01) when conditioned on the top genes, indicated by red dots (F). To assess the false-positive rate among the derived relationships after multiple tests, Monte Carlo simulation was done. The estimated false-positive rate for the 15 genes and one EST inferred to mediate PPARα–AILH after testing all of the top and bottom gene pairs is 0.002.

Specifically, we used LCA to identify gene pairs in which one was conditionally independent of *Ppara* when the other was conditioned on *Ppara* (Figure 3). When conditioned on the identified top genes, the correlation between the bottom gene and siRNA effect, manifested by the mRNA level of *Ppara,* vanished (Figure 3A, red dots). However, when conditioned on the bottom genes, the correlation between *Ppara* and the selected top genes remained significant (Figure 3B, red dots). Among approximately 25,000 genes profiled, 5,650 transcripts regulated by *Ppara* siRNA in at least one mouse liver (*p* < 0.01 revealed by a platform error model) were tested for potential pairwise relationships by LCA (Table S1). A total of 22,256 gene pairs that satisfied all of the six criteria specified in Materials and Methods were selected as the top–bottom, or upstream–downstream, gene pairs (*X* → *Y*) from the mouse expression profiles. These gene pairs with inferred regulatory relationships accounted for about 0.07% of all potential pairs among the 5,650 transcripts tested.

### Deriving Genes Mediating PPARα–AILH from a Rat PPAR Liver Minicompendium

In the second step of our approach, we inferred the causal genes for liver hypertrophy induced by PPARα activation by using the derived regulatory relationship within these gene pairs. Of the 5,650 mouse transcripts tested, 2,569 had reciprocal orthologs on the rat chip used for profiling the liver responses to PPARα agonists (Table S2). A total of 4,727 gene pairs with inferred regulatory relationships were identified among the 2,569 mouse genes with rat orthologs. Similarly, these gene pairs represented 0.07% of all potential pairs among the 2,569 genes. Assuming that the regulatory relationships derived in the mouse liver hold in the rat liver, we selected gene pairs only if there existed conditional independency between gene *X* and hypertrophy given gene *Y* in the PPAR minicompendium [29] and not conditional independency between gene *X* and gene *Y* given hypertrophy (Figure 3D–3F). Specifically, for all of the selected gene pairs, the dependency between the top genes and liver hypertrophy was abolished when conditioned on the bottom genes (*p* < 0.01) (Figure 3D, red dots). The dependency between the top genes and bottom genes remained when conditioned on liver hypertrophy (Figure 3E). So did the dependency between liver hypertrophy and the bottom genes (*p* < 0.01) when conditioned on the top genes (Figure 3F). The *Y* genes in the selected gene pairs were identified as the causal genes for PPARα–AILH if they occurred in at least one gene pair. We identified 39 transcripts as candidates most likely mediating PPARα–AILH (*p* < 0.01), and 16 of these transcripts—corresponding to 15 genes and one expression sequence tag (EST)—were significant at the level of *p* < 5 × 10^−4^ (Table 1).

**Table 1:**
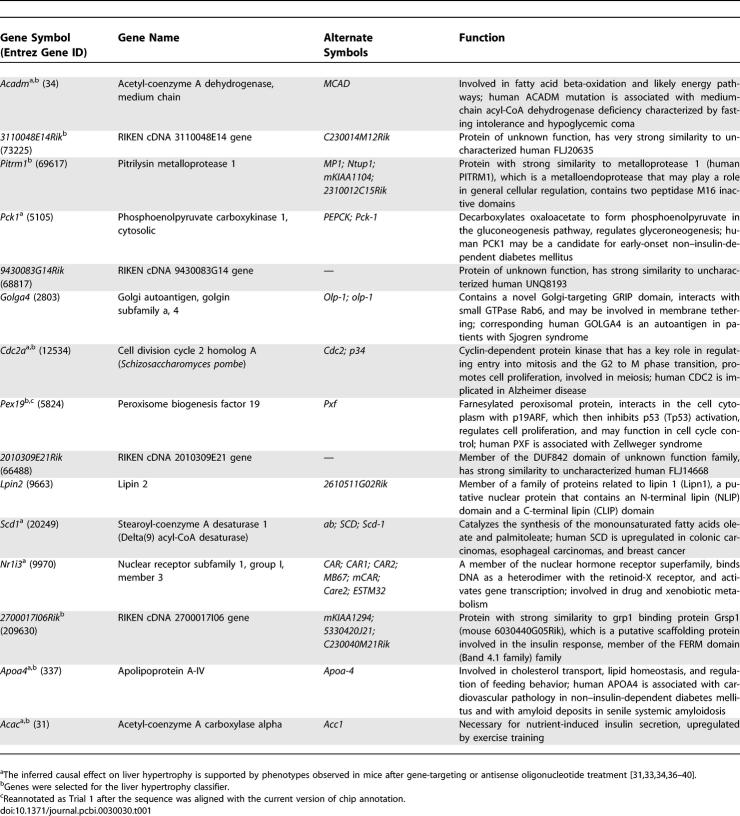
Fifteen Genes Inferred as Mediating PPARA–AILH and Their Functional Annotations

To check for false-positives among the derived relationships after multiple tests, we conducted a Monte Carlo simulation. The false-positive rate was estimated based on 1,000 simulations testing all 4,727 gene pairs. The chance of false discovery for the inferred causal genes mediating the PPARα–AILH was 0.002.

To validate our assumption that the regulatory relationship between gene pairs inferred in the mouse was retained in the rat, the conditional independencies between PPARα activity (measured by PPARα direct targets) and bottom genes given top genes for all 16 identified transcripts were examined in the rat liver PPAR minicompendium. Nine (56%) of the 16 transcripts satisfied the statistical tests. The remaining seven transcripts may have been excluded as a consequence of the different criteria used for measuring PPARα perturbation between the mouse and rat studies. For example, in the mouse study, PPARα activity was measured directly by the mRNA level in siRNA profiles, whereas in the rat study, it was measured indirectly by the mRNA levels of the PPARα target genes. Nevertheless, 11 of the 15 identified genes have comprehensive functional annotations, and eight of these 11 genes (73%) have been reported either to be associated with or to cause liver hypertrophy or hepatomegaly [30–32].

### Verifying the Inferred PPARα–AILH Causal Genes by Their Capability to Predict Non-PPARα–Induced Liver Hypertrophy

**Figure 4:**
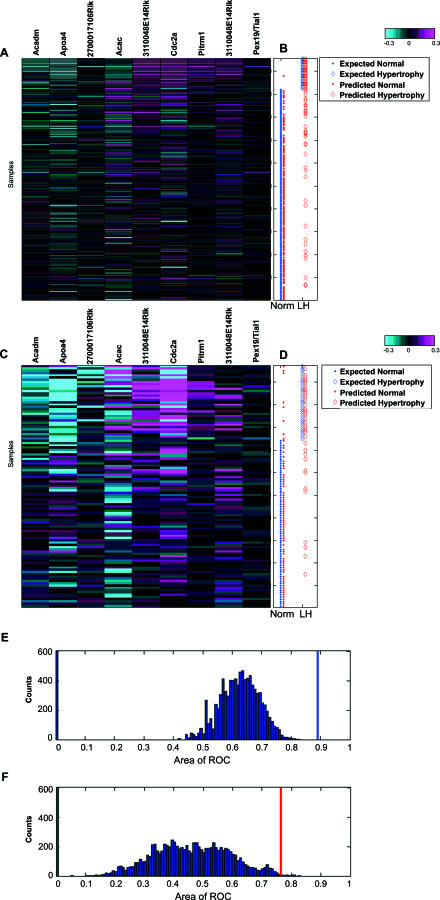
Predictability of Liver Hypertrophy Induced by Non-PPARα Compounds from Inferred PPARα–AILH Causal Genes Nine selected transcripts for the logistic regression model from the 16 inferred causal genes were coordinately regulated in the training set (A). A total of 211 expression profiles for the nine measured transcripts were aligned with liver hypertrophy, defined by the liver/body weight ratio (B) in corresponding rats (color bar: −0.3:0.3 at log_10_ scale). The expected normal liver and liver hypertrophy (LH), based on measured liver/body weight ratio in the training set, are indicated by blue dots and diamonds, respectively, while the predicted normal liver and LH derived from the established model are indicated by red dots and diamonds, respectively (B). Based on the logistic regression model built from the training set, the pathological condition of the liver in the independent testing set (normal or hypertrophy) was predicted (D). The expected normal liver and LH based on measured liver/body weight ratio in the testing set are illustrated by blue dots and diamonds, respectively. The predicted normal liver and LH derived from the established model from the training set are indicated by red dots and diamonds, respectively. Distinctive patterns of the selected biomarkers are evident between the normal and hypertrophic livers [(A,C); color bar: −0.3:0.3 at log_10_ scale]. The probability of obtaining such a set of predictive biomarkers from the genes correlated with the liver/body weight ratio in the PPAR minicompendium was significantly small in both the training dataset [(E), *p* < 0.001] and the testing dataset [(F), *p* < 0.005]. The AUCs for the ROC of the built model based on causal transcripts are indicated by the blue and red bars for the training and testing dataset, respectively, among the distribution of AUCs for 10,000 trials of 16 genes randomly selected from 757 genes correlated with the endpoint in the minicompendium (E,F).

We assumed that liver hypertrophy is mediated by a common essential molecular mechanism, even if it can be induced by differing top signals. Therefore, there should be a set of core genes that are essential for liver hypertrophy regardless of whether the hypertrophy results from PPARα activation or other conditions. If some of the 16 identified transcripts are essential for liver hypertrophy, we would expect them to have predictive power for compound-induced liver hypertrophy regardless of the compounds' mode of action; otherwise, the derived causal effect of these 16 transcripts for liver hypertrophy cannot be sustained. To test this hypothesis, we profiled 211 rats that were treated with 30 non-PPAR compounds and determined their liver/body weight ratio, as an indicator of liver hypertrophy (Table S3). A logistic regression-based classifier for liver hypertrophy was built based on nine of the 16 identified causal transcripts from the 211 profiles as the training set. The optimal number of transcripts among the 16 inferred transcripts was determined by their contribution to the fit of the model for predicting liver hypertrophy. The accuracy of the model was measured by the root-mean-squared error estimated by leave-one-out validation. Nine transcripts were selected to build the best-fitted model with the least difference between the measured and predicted ratios of liver to body weight in the training set. The built model predicted liver hypertrophy in the training set with 96% sensitivity and 80% specificity (Figure 4B, Table 2).

**Table 2:**
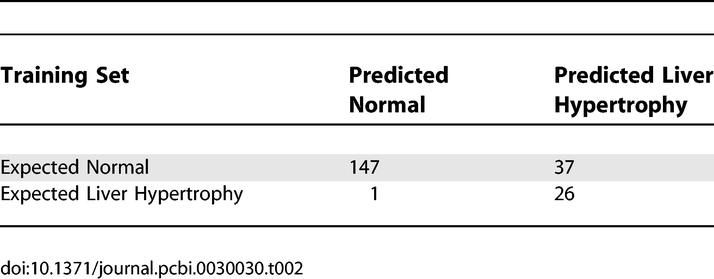
Estimated Optimal Performance of the Logistic Regression Model in the Training Set (*n* = 211 Profiles)

To further validate this model, we assessed its performance in an independent testing dataset. This dataset consisted of the nine genes and liver/body weight ratios from another set of 107 rat liver profiles obtained in response to treatment with 17 non-PPARα compounds (Table S4). These compounds have mechanisms of action or targets that differ from those of the 30 non-PPARα compounds used in the training set. The nine-gene model built in the training set achieved 84% sensitivity and 76% specificity in predicting liver hypertrophy in the independent testing set (Figure 4C and Table 3). This finding suggests that we can predict liver hypertrophy based on this model with an acceptable level of confidence regardless of the type of top signals that induce the liver hypertrophy.

**Table 3:**
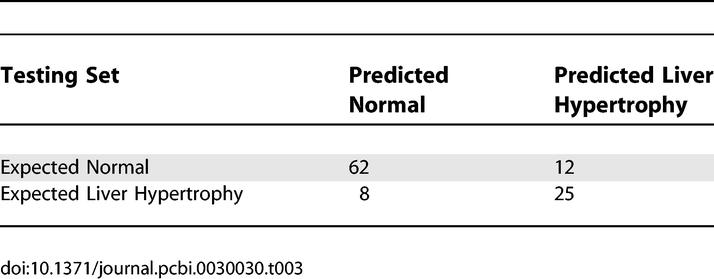
Estimated Performance of the Logistic Regression Model in the Independent Testing Set (*n* = 107 Profiles)

Of note, the selection of the 16 candidate transcripts on which the model was built was not dependent on either of the datasets used for training and testing. To determine if the causal information is necessary for such performance in predicting liver hypertrophy, we examined the possibility of achieving an equivalent level of accuracy in predicting liver hypertrophy based on genes selected solely by their association or correlation with the liver/body weight ratio in the PPAR minicompendium. Based on correlation with liver/body weight ratio, we identified 757 genes that had a value equal to or greater than that of the 16 inferred causal transcripts in the PPAR minicompendium. The identified genes were used as a candidate pool to build a correlation-based model. In total, we built 10,000 models based on 10,000 sets of 16 genes randomly selected from the candidate pool. Using the area under the curve (AUC) from a receiver operating characteristic (ROC) plot (a common indicator for model performance), we estimated the distribution of the performance for these 10,000 models. This in silico simulation revealed that none of the models in the training set, and only 37 of the models in the testing set, achieved a level of performance at least equal to that of the model based on inferred causal genes in predicting liver hypertrophy. The probability of building a model with at least the same level of performance as that built from the inferred causal transcripts was exceedingly small for both the training set (*p* < 0.001, Figure 4E) and the testing set (*p* < 0.005, Figure 4F). Hence, correlation alone is insufficient for extrapolating the capability of the 16 inferred PPARα–AILH causal transcripts to predict liver hypertrophy induced by non-PPAR compounds. The predictive power of the inferred PPARα–-AILH causal transcripts for non-PPAR–induced liver hypertrophy supports the assumption that essential biological components for liver hypertrophy exist. The inferred causality relationship between the 16 transcripts and liver hypertrophy is essential for the extrapolation of this prediction model.

### Inferring the Sufficiency Order among the Derived Causal Genes for Liver Hypertrophy

**Figure 5:**
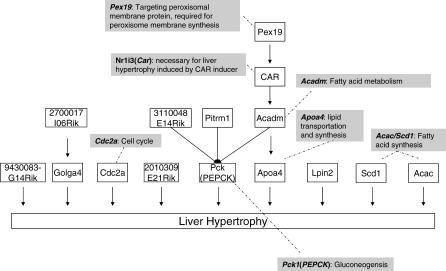
The Sufficiency Order Network of the 15 Causal Genes Mediating PPARα–AILH, as Determined by CI Tests against Liver Hypertrophy

Finally, we determined the sufficiency order for mediating PPARα–AILH among the 16 transcripts by exhaustive pairwise conditional independent (CI) tests (Figure 5 and Figure S1). There were 15 genes with known or predicted proteins among the 16 derived causal transcripts. CI tests identified nine of these genes as the most sufficient ones in inducing liver hypertrophy. For instance, as revealed by conditional dependency between *Pck1* and PPARα–AILH given the constraint of the inferred causal relationship between *Acadm* and PPARα–AILH, *Pck1* is more sufficient than *Acadm* in mediating PPARα–AILH (Figure S1A).

This order is also in agreement with observations in *Pck1* null mice and *Acadm* null mice. *Acadm,* also called medium-chain acyl-CoA dehydrogenase (MCAD), encodes an essential enzyme for fatty acid oxidation. MCAD deficiency in mice or humans can cause liver hypertrophy and hepatic steatosis [32,33]. Meanwhile, *Pck1,* also known as phosphoenolpyruvate carboxykinase (PEPCK), encodes a rate-limiting enzyme for gluconeogenesis. PCK1 deficiency is lethal in both mice and humans [34,35]. However, liver hypertrophy and steatosis have been observed in liver-specific PCK1-deficient mice [34]; after 14 hours of fasting, such mice have a 71% increase in liver weight along with significant hepatic lipid accumulation. Furthermore, although these mice have elevated expression of MCAD, this does not reverse the gain in liver weight induced by PCK1 deficiency. Similarly, an exhaustive search over all potential gene pairs between *Pck1* and the other inferred causal genes for PPARα–AILH revealed that *Pck1* is more sufficient in mediating liver hypertrophy than are *Pex19, Car, Pitrm1,* and *3110048E14Rik*.

Based on our knowledge or lack of knowledge about their functions, the nine genes derived to be most sufficient in mediating liver hypertrophy represent four groups: a fatty acid, lipid genesis, or energy metabolism group, including *Pck1, Apoa4, Scd1, Acac,* and *Lpin2;* a cell proliferation group, *Cdc2a,* also known as *cdk1;* a Golgi group, *Golga4;* and a group with unknown function, *2010309E21Rik* and *9430083G14Rik*. Five of these genes, *Pck1, Apoa4, Scd1, Acac* and *Cdc2a,* have been reported to be modified by gene-targeting or antisense oligonucleotide treatment, and in all five cases the resulting phenotypic alterations in the liver are consistent with the gene's inferred causal effect on liver hypertrophy as determined by our method [31,33,34,36–40].

In a final analysis, we examined potential reduction of statistical power associated with multiple tests for the genes with the maximal number of downstream genes mediating PPARα–AILH. For the nine genes that exhibited the highest sufficiency in mediating liver hypertrophy, the estimated probability that an additional causal gene from the rest of the 16 transcripts could be more sufficient for the hypertrophy was *p* = 0.07.

## Discussion

Overall, our genome-scale approach provides a powerful tool for unveiling components of potential pathways and the order of their sufficiency relationships in mediating a drug-induced toxicity. In selecting an example, we applied our approach to identify the key molecular components and their sufficiency order in PPARα–AILH. The proposed 15 causal genes not only reveal the molecular mechanism of this phenomenon, which is in agreement with that shown in previous studies, but also suggest potential unknown common mechanisms underlying liver hypertrophy. The inferred causality hypotheses orientated to a certain type of toxicity, in this case, liver hypertrophy, are essential in discovering the potential mechanism of drug-induced toxicity.

### Facilitating De Novo Discovery of Pathways in Rodents with RNAi Treatment Targeting a Single Gene

Computational approaches for de novo discovery of regulatory networks have been previously reported [14–20]. A few have attempted to associate the derived network with specific biological outcomes [18,22–24]. Nevertheless, application of these approaches in rodents is impeded by their requirement for a large number of targeted genetic perturbations or prior knowledge of the pathway. Our approach overcomes such hindrance by taking advantage of the siRNA off-target effects as additional genome-wide perturbations. Using the mRNA level of an siRNA-targeted gene, in the current case, *Ppara,* as the instrumental variable, in the siRNA-treated expression profiles, we were able to infer pairwise regulatory relationships by a simple but well-established LCA originally developed for data mining [27].

Instead of approximating a global regulatory structure, we focused on the sufficiency order among the causal genes identified by the two-step relay approach. Taking advantage of the derived causal structure from the causal gene to the liver hypertrophy as local constraints, the searching space for the 16 derived transcripts was reduced from *O*(2*^g^*) to *O*(*g* ^2^), where *g* is the number of derived causal genes. In our case, the searching space was reduced by 272-fold. This dramatic reduction enabled us to exhaustively search for the sufficiency network, which was, otherwise, computationally expensive or simply infeasible. In contrast, most of the previously reported approaches require Monte Carlo Markov chain or other sampling techniques to search for a local optimal network as an approximation of the global optimal structure.

Moreover, the derived pairwise regulatory relationships from siRNA expression profiles can also benefit most of the currently used computational approaches for pathway de novo discovery. For example, application of these relationships as local constraints in Monte Carlo Markov chain–based Bayesian methods will reduce the searching space and consequently improve the chance that any resulting local optimal structure is the real global solution.

The two-step relay strategy offers unprecedented flexibility, although the assumption of retained pairwise relationships among the gene pairs derived from the first step is essential and needs to be experimentally examined. Because the derived regulatory relationship between the top (upstream) gene and the bottom (downstream) gene reflects a transcriptional regulatory relationship within a gene pair, these relationships are independent from the instrumental signal, in our case, *Ppara.* As a result, such regulatory relationships can be used to infer causal genes for any phenotypic changes induced by compounds or other types of perturbation. This portability of the inferred regulatory relationship within gene pairs is extremely helpful for toxicogenomics study because the majority of toxicity studies, including gene expression profile studies, are performed in rats, whereas knockout or RNAi silencing is most commonly undertaken in mice.

In this work, we used perturbation introduced by siRNA targeting one gene. It is conceivable that the coverage and reliability of pairwise relationship across the genome can be further improved if multiple siRNAs targeting different genes are applied. Meanwhile, other strategies can be applied to experimentally evaluate the accuracy of the inferred causal relationship. For example, genetic engineering of top genes in C. elegans or *Drosophila* can be used to verify the regulatory effect of top genes on bottom genes, the key assumption of our method. Monitoring the temporal relationship between the expression regulations of these inferred causal genes and the phenotypic outcomes in liver will enable further validation of the inferred causal network in rodents as well. Consequently, a genome-wide phenotype-oriented network can be inferred and tested in a relatively high throughput fashion.

### Exploring Differences among Classification, Causality, and Extrapolation

Classification does not prove a causal relationship. Yet, only a causal relationship will enable extrapolation of an established model into prediction. Hence, capability of extrapolation can be used as a test of the derived causal relationship. Many classifiers or gene sets, established by vigorous machine learning procedures, have been reported to describe the extent of drug-induced toxicity or the outcome of cancer with reasonable accuracy. However, they do not allow us to distinguish whether these identified gene sets have a functionally important role in mediating those observed outcomes or are merely indicators for the underlying propensity of those outcomes. The performance of such types of classifiers for predicting drug-induced toxicity will be affected by the diversity of compounds used in the training set and testing set. When toxicity has to be predicted for compounds affecting targets different from the targets affected by compounds in the training set, the performance of a classifier not based on causality information will deteriorate dramatically. In other words, a classifier that is not based on causality will rarely have the capability of extrapolation. Our comparison between classifiers based on genes selected by inferred causality and by correlation alone demonstrates that causality information is necessary for compound-independent extrapolation in toxicity prediction (Figure 4). The performance of classifiers built on genes selected from the 757 genes correlated with liver/body weight ratio in the PPAR minicompendium became substantially inferior when these classifiers were used to predict liver hypertrophy induced by non-PPAR compounds. Our simulation indicates that classifiers based on correlated genes alone are unlikely to successfully predict or extrapolate drug-induced toxicity independently from the mode of action of compounds used in training set (*p* < 0.005). In contrast, the classifier we built based on inferred causal genes can predict liver hypertrophy independent from compounds' modes of action in the original training set. The capability of extrapolating a model that was initially built on PPAR compounds into predicting liver hypertrophy regardless of a compound's primary targets has not been demonstrated in any previous reports and has not been examined for classifiers based on association markers.

The legitimacy of using the capability of extrapolation as an analytic validation approach for the inferred causal genes is further supported by experimental evidence for the inferred 16 causal transcripts in in vivo models [31,33,34,36–40]. More than 85% (six of seven) of the inferred causal genes that have been genetically modified or specifically targeted in in vivo models are associated with phenotypic abnormalities in the liver that are in agreement with the role of these genes in mediating liver hypertrophy (Table 1). Five of them are among the genes with highest sufficiency in causing liver hypertrophy. In contrast, the rate of discovering functionally related genes among the top-ranked markers in an association-based classifier is quite low. For instance, Furey and colleagues detected only three genes that are cancer-related among the top ten most discriminative genes or ESTs for classifying ovarian cancer [41]. The rate for cancer-related genes or ESTs may be underestimated because they exclude noncoding sequences, which have been recently shown to play an important role in tumorigenesis. Nonetheless, the lack of evidence for the capability of extrapolating their association-based model into predicting outcomes of other solid tumors is in agreement with the contention that causality is a prerequisite for the capability of extrapolation in predicting outcomes.

### Evaluating Statistical Power and Assumptions in the Two-Step Relay Approach

Although we have made every effort to analytically validate the derived results of causal relation in general, several factors may affect our results. First, the assumption that the derived pairwise regulatory relationship between a top gene and a bottom gene is valid in both mouse and rat liver may not hold all the time. To gain confidence in this assumption, we examined the statistical conditional independence between PPARα activity, measured by direct targets of PPARα, and bottom genes given top genes for all 16 identified transcripts using the PPAR minicompendium. We found that nine of the 16 transcripts were more likely to retain the same top–bottom relationship as derived from the siRNA profiles. Of note, the original 16 gene pairs were established by treating the mRNA level of PPARα as a direct measurement of perturbation on *Ppara* in siRNA-treated profiles, whereas the nine gene pairs were reestablished by treating mRNA levels of *Cpt1a* and other genes known to be PPARα downstream genes as an indirect measurement of perturbations on *Ppara* in the PPAR minicompendium. Second, statistical power could be reduced with multiple tests. For example, false-positives may occur among the inferred causal genes for PPARα–AILH after testing all the 4,727 gene pairs. The estimated false-positive rate for the 16 identified transcripts was 0.002. Third, a measurement error associated with expression profiles could affect the sensitivity and specificity of our analysis. To account for this error, we used error-weighted mean logarithmic ratios in our analysis. Cross-validation from different platforms or independent experiments will help further address this issue.

### Uncovering Distinguishing Molecular Mechanisms Underlying PPARα–AILH

The five identified most sufficient causal genes for PPARα–AILH suggest that three major molecular mechanisms underlie the increases in liver weight mediated by this receptor: increased cell proliferation, altered fatty acid or lipid metabolism, and affected function of the Golgi apparatus. In agreement with observations in humanized PPARα mice treated by PPARα ligand Wy-14643, our results suggest that *Cdc2a (cdk1),* a cell cycle control gene, may increase liver weight by enhancing cell proliferation. As shown by Gonzalez and colleagues, replacing murine PPARα with human PPARα dramatically decreases the number of proliferating cells and inhibits the level of *cdk1* in the presence of Wy-14643 [37]. Although they clearly demonstrated the rodent specificity for the effect of PPARα activation in cell proliferation and tumorigenesis, the downstream genes mediating the proliferation effects of Wy-14643 remained largely unknown [37,38]. Our study provides a testable hypothesis for the role of *Cdc2a* or *cdk1* in mediating the rodent-specific tumorigenic effect of PPARα activation. A cross between humanized PPARα mice and liver-specific *Cdc2a* knockout mice will be a perfect model to evaluate the hypothesized contribution of *Cdc2a* to PPARα activation–induced cell proliferation and tumorigenesis.

The contribution of fatty acid or lipid metabolism to PPARα–AILH, suggested by our observations, is also supported by phenotypic changes reported in several genetically modified mouse models, including the mice with humanized PPARα. For instance, both *Acadm,* a gene critical for fatty acid metabolism, and the extent of liver hypertrophy, measured by either the absolute liver weight or the liver/body weight ratio, are increased by PPARα ligand Wy-14643 in mice with or without humanized PPARα despite the fact that cell proliferation is dramatically reduced with the humanized receptor PPARα [37,38]. Furthermore, for inferred causal genes downregulated by PPARα ligands, such as *Pck1* and *Apoa4,* knocking out these genes results in significant increases in liver weight and liver/body weight ratio or in fatty liver [34,39]. The increased liver weight or size resulting from deficient mutations of these two genes, and the fact that PPARα activation reduces their expression, supports their inferred role in liver hypertrophy. Additional evidence comes from the response to the high-fat diet, the endogenous ligand for PPARα, in mice treated with an antisense oligonucleotide for *Acac1* and *Scd1* [36,40]. As the key regulators for fatty acid oxidation and fat synthesis in the liver, *Acac1* and *Scd1* are upregulated by PPARα activation as well as by a high-fat diet. Increased expression of these genes results in increases in liver size or weight by lipid accumulation in hepatocytes. Antisense oligonucleotide inhibitors of *Acac1* and *Acac2* totally reverse this induced lipid accumulation, suggesting that *Acac1* mediates the effect of PPARα activation on lipid accumulation in liver [40]. Similar results have been reported in mice treated with an antisense oligonucleotide inhibitor of *Scd1* [36]. Injection of 15 mg/kg of this oligonucleotide twice a week significantly reduced the lipid accumulation in the liver induced by a high-fat diet. Taken together, these reported observations are not only in concert with our inferred role of fatty acid metabolism and lipid synthesis in mediating PPARα–AILH, but may also reveal a potential key pathological change, lipid accumulation, linking liver hypertrophy with fatty liver.

The role of *Golga4* in mediating PPARα–AILH remains unknown because functional studies of this gene are lacking. However, the essential role of Golgi traffic in regulating the activation of sterol regulatory element–binding protein (SREBP), another key regulator of lipid metabolism, points in the same direction.

Distinguishing the proliferation component from the fatty acid and lipid metabolism component for liver hypertrophy is important for drug development. For example, compounds are being developed to target *Pck1* as novel therapeutic agents for type II diabetes or obesity. Distinguishing the two components enables us not only to predict the potential effect on liver weight by compounds directly targeting *Pck1* (Pepck), a potential therapeutic target regulated by PPARα and the rate-limiting enzyme for gluconeogenesis, but also to monitor any cellular proliferation associated with increased liver weight.

### Revealing Common Essential Molecular Machinery That Mediates Liver Hypertrophy Shared by a Variety of Compounds with Different Mechanisms of Action

The causal sufficiency order among the 15 derived genes suggests that liver hypertrophy induced by compounds with drastically different mechanisms of actions may be mediated by common molecular components. For example, we found that *Car,* another nuclear receptor whose activation is also known to cause liver hypertrophy [31], was more sufficient than *Pex19,* a gene required for the synthesis of the peroxisome membrane, in mediating PPARα–AILH. To our knowledge, the effect of PPARα ligands on the liver in mice with a *Car*-deficient mutation has never been reported; however, liver hypertrophy induced by PPARα activation and by *Car* activation might share the same mechanism, given the extensive crosstalk between PPARα and *Car* ligands and their very similar effects on some of the genes regulating fatty acid or lipid metabolism. Finally, the capability to extrapolate a model built on causal genes for PPARα–AILH to predict liver hypertrophy induced by a set of non-PPAR compounds with different mechanisms of action supports the existence of common essential molecular machinery that mediates liver hypertrophy independent of upstream signals.

In conclusion, our approach enables systematic inference of testable hypotheses about potential molecular mechanisms underlying drug-induced toxicity. The validity of any inferred hypotheses will still be subject to experimental verification. Nonetheless, the derived causal sufficiency order provides a way to prioritize all these hypotheses, which otherwise cannot be tested exhaustively across the prohibitive number of potential interactions and combinations among the genes associated with drug-induced effects.

## Materials and Methods

### *Ppara* siRNA treatment and expression profiles in the mouse.

Two different siRNAs targeting *Ppara* and two control siRNAs targeting secreted alkaline phosphatase (SEAP) and GL3 were delivered to the mouse liver by hydrodynamic tail vein injection [25]. Four animals per group were treated 48 hours before livers were collected. RNAs were extracted and profiled on a custom-designed mouse oligonucleotide microarray with approximately 25,000 genes according to a published procedure [29]. In total, the *Ppara* siRNA mouse expression profiles included eight profiles for the *Ppara* siRNAs targeted to two different fragments of the *Ppara* mRNA sequence, four profiles for the SEAP control siRNA, four profiles for the GL3 control siRNA, and eight profiles for a vehicle control (Ringer solution).

### Liver PPAR minicompendium and liver responsive datasets in the rat.

In brief, the PPAR minicompendium used in this study consists of expression profiles of livers from 147 rats treated with activators of PPARs and other nuclear receptors, including the PPARα agonists fenofibrate (400 mg/kg per day; Sigma-Aldrich, http://www.sigmaaldrich.com) and Wy-14643 (100 mg/kg per day; ChemSyn Sciences, http://www.chemsyn.com), the PPARγ agonist rosiglitazone (100 mg/kg per day), the pan-PPAR agonist bezafibrate (250 mg/kg per day; Sigma-Aldrich), and the retinoic acid receptor agonist all-*trans* retinoic acid (25 mg/kg per day; Sigma-Aldrich). Female Sprague Dawley rats (Charles River Laboratory, http://www.criver.com) 7–8 wk of age were dosed daily for 2, 3, 4, 5, and 6 days by oral gavage. Six hours after the last dose, animals were euthanized by isoflurane inhalation followed by blood collection from the vena cava and exsanguination. Liver weight and body weight were measured for all animals, and the ratio of liver to body weight was used as the index for liver hypertrophy. RNAs were extracted from liver and profiled on a custom-designed rat oligonucleotide microarray with approximately 25,000 genes according to a published protocol [29]. The error-weighted ratios for the 2,569 transcripts used in our study are summarized in Table S2. A similar study was conducted using 318 liver samples from rats treated with 47 compounds whose primary targets were not PPARs, hereafter referred to as non-PPAR compounds. Data used in our analyses are summarized in Table S3. All the animals used in these experiments were handled in laboratories accredited by the Association for Assessment and Accreditation of Laboratory Animal Care International. The animal studies were conducted with Institutional Animal Care and Use Committee approval. A comprehensive summary of related data and analyses for this PPAR rat liver minicompendium were reported separately [29].

### Local constraint algorithm analysis for inferring causal regulatory relationships from siRNA-treated expression profiles.

The three causal models corresponding to the major scenarios for the impact of siRNA on other genes and cellular responses (Figure 2) share a common regulatory relationship between *X* and *Y* (denoted as *X* → *Y*). Cooper [27] and Spirtes and colleagues [28] have demonstrated that when *W* is an instrumental variable, or when *W* is not caused by any measurable variables, such as *X* and *Y,* the causal relationship between *X* and *Y* can be inferred by a constraint-based causal discovery method. Specifically, when six dependent and independent constraints among *W, X,* and *Y* hold—(1) D(*W, X*), representing dependence between *W* and *X,* (2) D(*X, Y*), (3) D(*W, Y*), (4) D(*W, X* given *Y*), representing dependence between *W* and *X* when conditional on *Y,* (5) D (*X, Y* given *W*), and (6) I (*W, Y* given *X*), representing independence between *W* and *Y* when conditional on *X*—it was sufficient to identify the causal relationship *X* → *Y* from 96 possible regulatory models among the triplet *W, X,* and *Y* elicited by Cooper [27]. Because the mRNA levels of siRNAs' on- and off-target genes are presumably controlled by the siRNA, the mRNA level of the siRNA-targeted genes could be considered a good measure for the on-target effect of the siRNA. Therefore, the siRNA can serve as the instrumental variable (*W*). Furthermore, it was assumed that the relationship between *X* and *Y* was not confounded by *W* or any hidden process in the possible scenarios for the impact of the siRNA. Therefore, those pairwise causal relationships between a top gene (*X*) and a bottom gene (*Y*), or a causal gene (*X*) and its cellular response (*Y*), affected by siRNA targeting a single gene can be inferred by LCA from expression profiles on the genome scale [27].

### A two-step relay method for inferring genes mediating PPARα–AILH from the PPAR minicompendium.

Genes mediating PPARα–AILH were inferred by a two-step relay method as illustrated in Figure 2G. In the first step, the pairwise causal relationship (*X* → *Y*) between genes was inferred by LCA from the expression profiles obtained in response to *Ppara* siRNA treatment. In the second step, the derived relationship *X* → *Y* was relayed to identify the local causal structure among the top gene (*X*), the bottom gene (*Y*), and the phenotypic response (*R*), liver hypertrophy. The top gene (*X*) was then considered as the instrumental variable *W,* the bottom gene (*Y*) was considered as *X,* and the response (liver hypertrophy) was considered as *Y* in our causal model (Figure 2). Similarly, as specified by Cooper's LCA, when the six previously defined constraints hold, it is sufficient to infer the causal relationship *Y* → *R*, with which the bottom gene *Y* causes *R* (i.e., liver hypertrophy), in the second step from the PPAR minicompendium. The key assumption that permits the relaying is that the regulatory relationship, *X* → *Y,* within the gene pairs derived from the mouse liver expression profiles is valid in the rat liver expression profiles.

### Constraint-based method for determining a sufficiency order among causal genes.

The sufficiency order among causal genes (*Y, Y′, Y″, …*) was determined by a series of CI tests between two causal genes and the cellular response. Specifically, when two criteria are met—(1) a causal gene *Y* and a cellular response *R* are independent given another causal gene *Y′,* and (2) gene *Y′* is not independent with cellular responses conditioning on gene *Y*—gene *Y′* was considered to be more sufficient than gene *Y* in causing the cellular response *R*. By exhaustively examining all pairwise relationships among causal genes (*Y, Y′, Y″,* …), the sufficiency order network among all identified key genes can be established.

## Supporting Information

Figure S1 Determination of the Sufficiency Order among *Pck1, Apoa4,* and *Acadm* in Mediating PPARα–AILH by Conditional Dependency Testing Although regulation of both *Pck1* and *Acadm* is necessary for PPARα–AILH, these genes are not equally sufficient to mediate liver hypertrophy. As indicated by the partial correlation coefficient between *Acadm* and the liver/body weight ratio when conditioned on *Pck1, Acadm* is likely to be conditionally independent from liver hypertrophy given *Pck1* [(A), left, where the correlation drops to 0.1]. However, when conditioned on *Acadm,* the correlation coefficient between *Pck1* and the liver/body weight ratio remains at a level of 0.36 ((A), right). This difference suggests that *Pck1* is more sufficient than *Acadm* in mediating PPARα–AILH. The correlation coefficient between *Apoa4* and liver hypertrophy given *Acadm* is not significantly changed, implying that *Apoa4* is more sufficient than *Acadm* in mediating PPARα–AILH [(B), right].
(679 KB PDF)

Table S1 PPARα siRNA–Treated Mouse Liver Dataset (2.5 MB XLS)

Table S2 PPARα Rat Liver Dataset (6.9 MB XLS)

Table S3 Liver Hypertrophy Training Dataset (117 KB XLS)

Table S4 Liver Hypertrophy Testing Dataset (67 KB XLS)

### Accession Numbers

Gene ID numbers from the Entrez Gene database (http://www.ncbi.nlm.nih.gov/entrez/query.fcgi?db=gene) for the genes mentioned in this paper are: *Acac* (31), *Acadl* (33), *Acadm* (34), *Apoa4* (337), *Cdc2a* (12534), *Cpt1a* (1374), *Golga4* (2803), *Lpin2* (9663), *Nrli3* (9970), *Pck1* (5105), *Pex19* (5824), *Pitrm1* (69617), *Ppara* (5465), *Scd1* (20249), *2010309E21Rik* (66488), *2700017I06Rik* (209630), *3110048E14Rik* (73225), and *9430083G14Rik* (68817).

## Abbreviations

AILH: activation-induced liver hypertrophy
AUC: area under the curve
CI: conditional independent
EST: expression sequence tag
LCA: local constraint algorithm
MCAD: medium-chain acyl-CoA dehydrogenase
PPARα: peroxisome proliferator-activated receptor α
RNAi: RNA interference
ROC: receiver operating characteristic
SEAP: secreted alkaline phosphatase
siRNA: small interfering RNA
