# Supplementary material for: PPARα siRNA–Treated Expression Profiles Uncover the Causal Sufficiency Network for Compound-Induced Liver Hypertrophy
Source: PLoS Comput Biol. 2007 Mar 2;3(3):e30. doi: 10.1371/journal.pcbi.0030030 (PMC1808491; doi:10.1371/journal.pcbi.0030030)
Supplement: Supplementary file 1 [file pcbi.0030030.sg001.pdf]

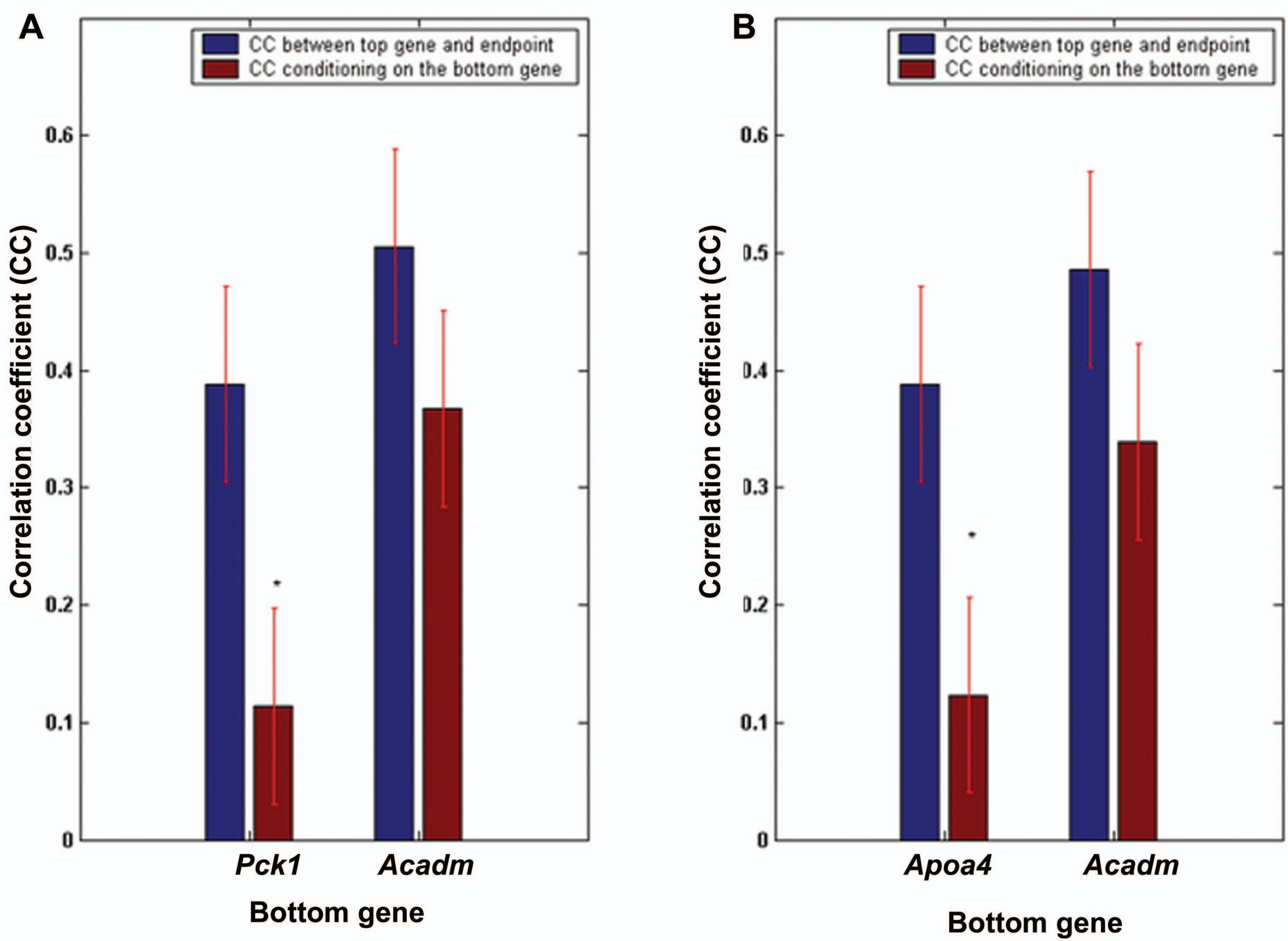

A
CC between top gene and endpoint
CC conditioning on the bottom gene
Correlation coefficient (CC)
0
0.1
0.2
0.3
0.4
0.5
0.6
*
Pck1
Acadm
Bottom gene
B
CC between top gene and endpoint
CC conditioning on the bottom gene
Correlation coefficient (CC)
0
0.1
0.2
0.3
0.4
0.5
0.6
*
Apoa4
Acadm
Bottom gene
